# Cancer impact on lower-income patients in Malaysian public healthcare: An exploration of out-of-pocket expenses, productivity loss, and financial coping strategies

**DOI:** 10.1371/journal.pone.0311815

**Published:** 2024-10-09

**Authors:** Farhana Aminuddin, Sivaraj Raman, Mohd Shahri Bahari, Nur Amalina Zaimi, Mohd Shaiful Jefri Mohd Nor Sham Kunusagaran, Nur Azmiah Zainuddin, Marhaini Mostapha, Tan Yui Ping, Nor Zam Azihan Mohd Hassan

**Affiliations:** 1 Institute for Health Systems Research, Centre of Health Economics Research, National Institutes of Health, Ministry of Health Malaysia, Shah Alam, Selangor, Malaysia; 2 Institute for Health Systems Research, National Institutes of Health, Centre of Health Policy Research, Ministry of Health Malaysia, Shah Alam, Selangor, Malaysia; Pharmaceutical Services Division, MALAYSIA

## Abstract

Cancer patients often grapple with substantial out-of-pocket (OOP) expenses and productivity loss, with the ramifications being particularly crucial for lower-income households. This study aims to estimate OOP costs incurred by cancer patients, assess their productivity loss, and analyse the financial coping mechanisms employed by individuals within the lower-income bracket. The study employed face-to-face interviews among cancer patients aged 40 years and above, currently undergoing treatment, and belonging to the lower-income group. Participants were recruited from six public cancer referral hospitals. OOP expenses, encompassing medical and non-medical costs, along with productivity loss, were measured. A generalized linear model was applied to identify potential OOP determinants. Additionally, the coping mechanisms employed by individuals to finance their cancer OOP expenses were also determined. Among the 430 participants recruited, predominantly female (63.5%), and aged 60 or older (53.9%). The annual mean total cancer costs per patient were US$ 2,398.28 (±2,168.74), including 15% for medical costs US$ 350.95 (±560.24), 34% for non-medical costs US$820.24 (±818.24), and 51% for productivity loss costs US$1,227.09 (±1,809.09). Transportation, nutritional supplements, outpatient treatment, and medical supplies were notable cost contributors to total OOP expenditures. Ethnicity (β = 1.44; 95%CI = 1.15–1.79), household income (β = 1.40; 95%CI = 1.10–1.78), annual outpatient visits (β = 1.00; 95%CI = 1.00–1.01), age (β = 0.74; 95%CI = 0.56–0.98), and employment status (β = 0.54; 95%CI = 0.72–1.34) were identified as significant predictors of OOP costs among cancer patients. Notably, 91% of participants relied on household salaries and savings, while 15% resorted to interest-free borrowing, 11% sold possessions, and 0.5% borrowed with interest to finance their expenses. This study offers crucial insights into the economic impact of cancer on individuals and their families, providing policymakers with valuable information to tackle challenges faced in their journey. Despite substantial public healthcare subsidies, the study revealed that cancer costs can remain a potential barrier to accessing essential treatment. Therefore, there is a need for reinforced system-level infrastructure to facilitate targeted financial navigation services.

## Introduction

Cancer, a widespread and devastating disease, affects millions of people worldwide, leaving a profound consequence on individuals and society as a whole [1]. The repercussions extend beyond health, affecting the finances and productivity of individuals. The impact is particularly pronounced in low- and middle-income countries, where healthcare resources may be limited, and individuals may face challenges in accessing affordable and effective cancer care [2]. From an economic point of view, the burden of cancer is multifactorial, encompassing direct medical costs, productivity losses due to illness and premature death, and the costs of caregiving [3–5]. This financial burden is exacerbated for those with limited economic resources. Lower-income populations grapple with a distinct set of challenges in managing their healthcare costs, with OOP expenses emerging as a significant and often overwhelming aspect of the cancer journey [6]. The ASEAN Costs in Oncology (ACTION) study in 2012 for example reported almost half of the households with cancer patients experienced catastrophic health expenditure. This was particularly true for patients in advanced disease stages and those from socioeconomically disadvantaged groups [7].

Studies have consistently highlighted the disproportionate burden of OOP cancer expenses carried by lower-income individuals compared to their higher-income counterparts [6,8,9]. This situation worsens for those residing in rural and remote areas who have to spend more on transportation to access cancer treatment [10–13]. A study reported that 25% of the Malaysian population lives more than 100 km away from radiotherapy facilities [14]. This issue is particularly significant for rural patients, especially those in Sabah and Sarawak, who potentially be forced to travel long distances and struggle to afford transportation and lodging. Beyond the financial toll, there is a cascading effect on productivity. The intricate dynamics between cancer and productivity loss are well-documented, with cancer survivors facing unique challenges. Frequent medical appointments, treatments requiring time off work, and the overall physical and emotional toll of battling cancer contribute to heightened productivity loss and increased rates of bankruptcy [15]. Individuals burdened by OOP expenses and productivity loss may find it challenging to access essential healthcare services, potentially leading to unmet needs and worsening health outcomes. The identification of relevant costs impacting cancer patients is thus crucial for understanding and addressing the financial challenges they face. This knowledge can assist healthcare organizations in tailoring financial assistance programmes and support services to meet the unique needs of patients.

Malaysia forms an interesting case study of cancer OOP expenditures as its widely acknowledged universal healthcare is provided through a combination of the public-funded healthcare system and private facilities. Public healthcare services are particularly heavily subsidised for all Malaysian citizens, with only nominal fees applied to specific services. Despite the significant subsidies and minimal charges at government hospitals in contrast to private facilities, research indicates that the diagnosis of cancer and subsequent care potentially impose a substantial financial burden on the bottom 40% income group of households (B40) in Malaysia [16]. Despite the evident need for targeted support mechanisms, research on the financial burden of cancer remains in its infancy. This paucity of information poses a significant impediment to effective planning and execution of health interventions. Therefore, this study endeavors to fill this critical gap by meticulously estimating the costs incurred by cancer patients in the lower-income group. Through a comprehensive exploration of OOP expenses and productivity loss, this study also hopes to unravel the coping strategies adopted by these patients. The present study seeks to inform policymakers, healthcare providers, and researchers and pave the way for targeted interventions for mitigating the financial impact of cancer among lower-income individuals.

## Methods

### Study design and setting

A cross-sectional costing study was carried out between June and October 2022 to assess the cost incurred on cancer patients receiving treatment at six referral cancer hospitals funded under the Ministry of Health (MOH) Malaysia. The six hospitals are Penang General Hospital (Northern Region), National Cancer Institute and Kuala Lumpur Hospital (Central Region), Sultan Ismail Hospital (Southern Region), Sabah Woman and Children Hospital and Sarawak General Hospital (Eastern Region). All these sites serve a diverse multi-racial population, providing comprehensive oncology and radiotherapy services to patients in their respective regions.

## Study participants and sampling

The study included cancer patients from low-income households attending the study sites, diagnosed with any type of cancer, and actively undergoing treatment. Inclusion criteria comprised low-income households falling within the bottom 40% (B40) of the Malaysian population, earning below MYR 5,250 [17], and recipients of the *Sumbangan Tunai Rahmah* programme (STR). The STR is a government financial aid programme aimed at assisting the plights of vulnerable groups. Eligible participants were those currently on treatment, aged 40 years and above, and capable of responding to the interview. The criterion of being aged 40 years and above was determined based on the rising incidence rates observed in all cancers combined [18]. Additionally, in cases of less verbal patients, their carers were also interviewed as long as they are able to provide detailed information of the cancer-related expenses. On the other hand, exclusions encompassed patient’s incapable of responding and with no companion and those solely on follow-up.

All eligible patients attending the oncology centre during the designated period were identified and recruited by convenience sampling. This non-probability sampling method was chosen because of the practical limitation of reaching a specific population of low-income cancer patients. Although convenience sampling does not provide the same level of generalizability as probability sampling, it was considered adequate for the research nature of this study.

### Sample size calculation

The calculated sample size, determined using the formula for a single mean. This to ensure a reliable estimate of the mean costs associated with cancer. Given the variability in costs, as reflected by the standard deviation observed in previous pilot study (*σ* = MYR 8902.24) [16], the margin of error (*E*) was set at 10% of the standard deviation, which correspond to MYR 890.22. This margin of error was chosen to achieve a balance between statistical precision and the feasibility of recruiting an adequate number of participants. Using the formula for sample size estimation: $n=(\frac{Z\times \sigma }{E}{)}^{2}$, where:

*Z* is the value corresponding to a 95% confidence level (*Z* = 1.96);

*σ* is the standard deviation of the costs within a population observed in the pilot study;

*E* is the margin of error, set at 10% of the standard deviation.

The required sample size was estimated to be 384 patients. Accounting for a potential 10% nonresponse rate, the study aimed to involve 427 cancer patients in its participation. This approach ensured that the study could achieve sufficient precision while accommodating the practical constraints of patient recruitment.

### Data collection method

The study employed a face-to-face interview using a structured and validated questionnaire, as detailed in the published pilot and feasibility study [16]. These questionnaires (see S1 Appendix) encompassed general sociodemographic and clinical profiles, cancer OOP expenses, costs associated with productivity loss (encompassing both paid work and unpaid household activities such as caregiving and housework), and details regarding financial coping mechanisms. Data were gathered rei during the interview, with patients were asked to recall their past expenses related to cancer care. Only expenses incurred within public healthcare facilities were included, while costs associated with private healthcare facilities were excluded. The operational definition of various costs was summarised in the Table 1.

**Table 1:** Glossary of the operational definitions of various costs variables.

Costs	Description
Medical costs	The expenses incurred as both an outpatient and inpatient, including costs for diagnostic tests, operations, primary treatments such as chemotherapy, and medical supplies. A 12-month recall period was used for inpatient services, while 3-month recall period was employed for other medical cost items.
Non-medical	Additional costs incurred by patients in accessing treatment, such as transportation, accommodation, meals, childcare, nutritional supplements, and alternative treatment. Transport costs included expenses for travel by bus, train, car (petrol, tolls and parking fees), and taxi fares to and from the hospital. A 3-month recall period was employed.
Productivity loss costs	The opportunity cost of an individual’s lost time due to illness and seeking treatment. Information regarding self-reported monthly income, number of days absent from work and days (in hours) of unable to do routine/ household tasks. A recall period of 1–2 months was employed.
OOP costs/ OOP expenses	The sum of medical and non-medical costs. Costs were annualized to determine one year cancer costs.
Total costs	The sum of OOP costs and productivity loss costs. Costs were annualized to determine one year cancer costs.

### OOP and productivity loss costs estimation

OOP expenses included the reported medical and non-medical costs, factoring in any reimbursements that were deducted. Medical costs encompass self-reported OOP expenses made for diagnosis and treatment. On the other hand, non-medical costs include expenses related to transportation, lodging, meal expenditure, childcare, nutritional supplements and alternative treatments incurred by the patients. For patients utilising personal transportation, the cost was computed by considering fuel consumption relative to the travel distance from their residence to the treatment site (MYR 0.50/km) [19]. In cases of alternative transportation modes, the calculation involved multiplying the reported transport fee by the number of trips. Both medical and non-medical cost components were collected over the three months preceding the interview and then annualized to determine the total OOP cost per year.

**Fig 1:**
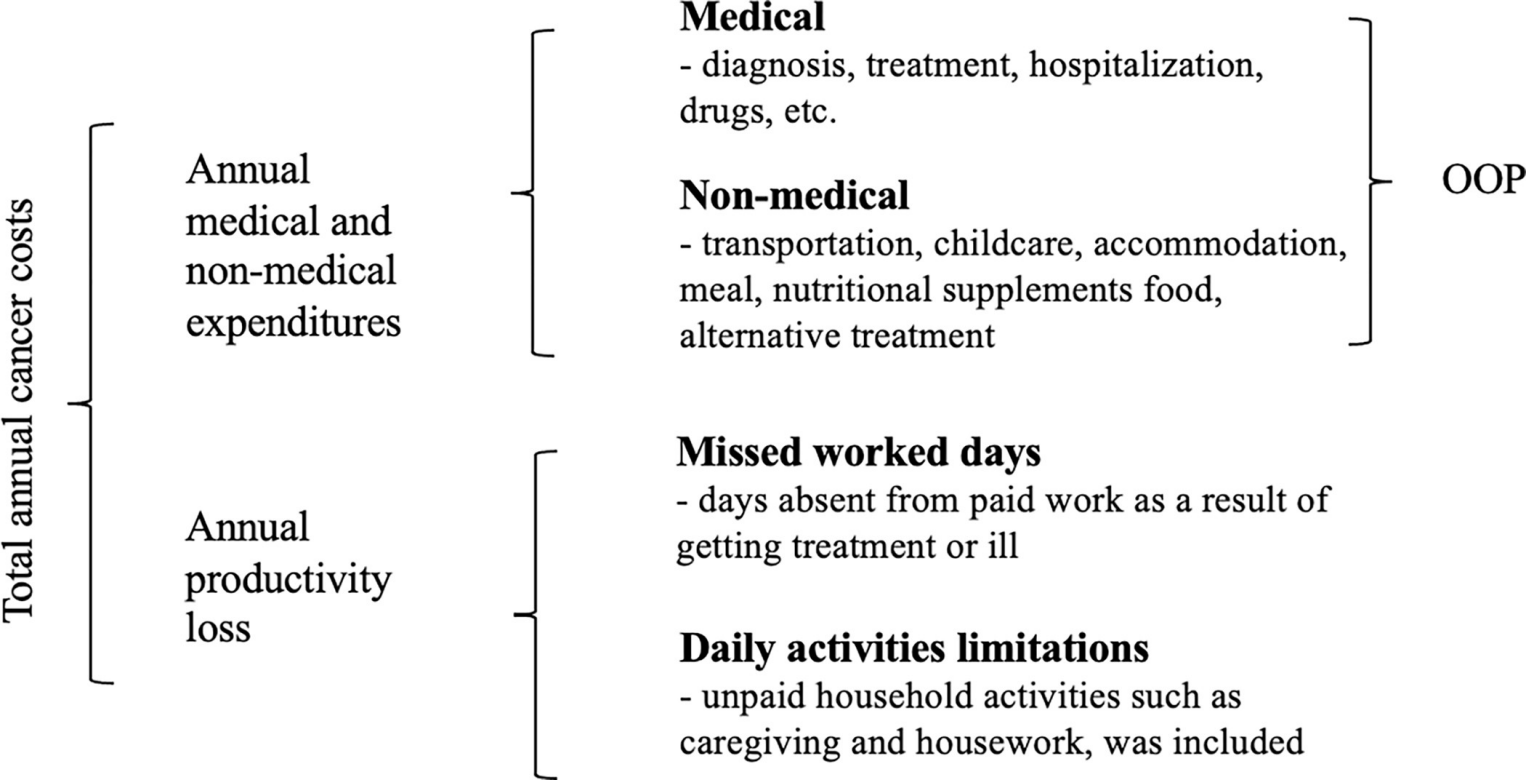
Overview of total cancer costs: The sum of annual OOP expenditure and productivity loss.

Productivity losses due to absenteeism, resulted from individuals missing work due to cancer, affecting both short-term and long-term work absences. The cost associated with productivity loss was estimated by utilising self-reported wages from patients who were currently employed or had any other source of income. The calculation involved multiplying the number of missed workdays by the daily wage, assuming each absent day equated to eight working hours. Additionally, health issues arising from cancer impede individuals from performing routine activities with genuine economic value, such as household work, childcare, or volunteer work [20]. The loss in ’production’ was either forfeited or taken on by others, who had to allocate scarce time that would have been otherwise spent on different activities. Some estimates of lost productivity incorporated unpaid productivity. To address this, the productivity loss for unemployed patients was determined using the National Minimum Wage Malaysia 2022, set at MYR1500.00. Furthermore, the productivity loss for elderly cancer patients aged 60 and above was calculated based on their estimated productive hours, which were reported as 6 hours [21]. A conceptual framework of total cancer costs, as depicted in Fig 1, underscores the financial burden faced by patients, encompassing medical, non-medical, and productivity loss.

### Financial coping strategies and distress financing

Incurring OOP expenses, affected cancer patients and their families utilised various financial coping strategies, including their income/savings; contributions from friends and relatives, borrowing with interest; borrowing without interest; selling physical assets; and any other available source. Distress financing was deemed to occur when a household had to resort to borrowing money or selling assets to meet OOP expenses [22–24].

### Statistical analysis

Descriptive statistics were reported for continuous variables either as mean (± standard deviation, SD) or median (interquartile range, IQR) while categorical variables were presented in frequencies (percentage, %). As cost data are often highly skewed, a nonparametric Mann-Whitney U test was applied to explore differences based on selected variables. Additionally, a generalized linear model (GLM) with a log link function and gamma distribution was used to discern the predictors influencing OOP expenditure [25]. GLM models utilising the gamma distribution were deemed the most fitting choice due to their ability to analyse both the mean and variance functions, effectively addressing the often rightly-skewed cost data [26–28]. The model featured OOP expenditure as the dependent variable, with possible independent variables spanning socio-demographics (age, gender, marital status, occupation, education, region, monthly household income, distance to cancer centre) and clinical profiles (cancer type, cancer stage, duration of cancer, number of inpatient visits, hospitalization days, and number of outpatient visits). Potential predictors with a *p*-value of less than 0.25 in univariate analysis were included in the final GLM. The goodness-of-fit of the GLM model was checked by applying the deviance, Pearson chi-square statistics and the likelihood ratio chi-square. All analysis was done using the commercially available statistical package for the social sciences, SPSS version 26.0 (SPSS Inc., Chicago, Illinois, USA), with the significance level set at two-sided α = 0.05. All costs were estimated for a one-year period, reported in Malaysian Ringgit (MYR), and, where applicable, presented in US dollars. The values were adjusted to 2023 levels using the exchange rate of US$ 1 to MYR 4.6295, as per the Bank Negara Malaysia Exchange Rates on 16 August 2023 [29].

### Ethics approval and consent to participate

The study obtained ethical approval from the Medical Research Ethics Committee (MREC) of the Ministry of Health Malaysia (KKM/NIHSEC/P21-1265). Written permissions were also obtained from each study site before commencing data collection. Before interviews, participants provided both written and verbal informed consent after being thoroughly briefed on the study’s objectives. Participants were assured of the strict confidentiality and privacy maintenance of the information they provided.

## Results

### Sociodemographic and clinical profiles

The sociodemographic details and clinical profiles of the 430 study participants are summarized in Tables 2 and 3. The majority (38.6%) of patients undergoing treatment fell within the age group of 60 to 69 years. A predominant proportion were female (63.5%), married (79.3%) and possessed a secondary level of education (57.7%). A significant number of patients were not currently in the workforce, consisting of those retired (n = 48, 11.2%) and unemployed (n = 336, 78.1%) individuals. Among the unemployed, approximately 30% had to quit their jobs due to cancer. The mean household size was 4.14 (2.04) members, with a mean monthly household income of MYR 3,211.39 (MYR 2,576.46).

**Table 2:** Sociodemographic profiles of study participants.

Variables	Category	Number	Percentage	Mean (SD)
Age (years)	40–49	64	14.9	59.84 (9.25)
	50–59	134	31.2	
	60–69	166	38.6	
	≥70	66	15.3	
Gender	Male	157	36.5	
	Female	273	63.5	
Ethnicity	Malay	174	40.5	
	Chinese	129	30.0	
	Indian	41	9.5	
	Indigenous Sabah	39	10.5	
	Indigenous Sarawak	45	9.1	
	Others	2	0.5	
Region	Peninsular Malaysia	298	69.3	
	Sabah and Sarawak	132	30.7	
Marital Status	Married	341	79.3	
	Divorced/ separated/ widow	55	12.8	
	Single	34	7.9	
Education Level	Primary level	120	27.9	
	Secondary level	248	57.7	
	Tertiary level	32	7.4	
	No formal education	30	7.0	
Employment	Employed	45	10.5	
	Unemployed	336	78.1	
	Retiree	49	11.4	
Unemployment due to cancer (n = 336)	Yes	102	30.4	
	No	234	69.6	
Household size	1–4	264	61.4	4.14 (2.04)
	5–8	153	35.6	
	≥9	13	3.0	
Monthly household income (MYR)	<2000	153	35.6	3211.39 (2576.46)
	2000–4500	180	41.9	
	>4500	97	22.6	
Distance to cancer centre (km)				71.96 (129.22)

**Table 3:** Clinical characteristics of study participants.

Variables	Category	Number	Percentage
Diagnosis (cancer site)	Breast	135	31.4
	Respiratory	48	11.2
	Gastrointestinal	106	24.7
	Urogenital	23	5.3
	Female reproductive	47	10.9
	Others	71	16.5
Cancer stage	Stage 1	18	4.2
	Stage 2	71	16.5
	Stage 3	139	32.3
	Stage 4	202	47.0
Present treatment	Chemotherapy	336	78.1
	Radiotherapy	57	13.3
	Surgery	10	2.3
	Chemotherapy + Radiotherapy	23	5.3
	*Others	4	0.9
Experience getting treatment at Private Facilities	Yes	39	9.1
	No	391	90.9
Time since diagnosis	≥1 year	203	47.2
	<1 year	227	52.8
Inpatient visits/ year, mean (SD)	1.68 (1.97)		
Length of stay/ year, mean (SD)	10.29 (12.30)		
Outpatient visits/ year, mean (SD)	30.11 (25.38)		

^*^Includes treatment other than chemotherapy, radiotherapy and surgery.

In terms of disease-related characteristics, approximately 31.4% of patients were diagnosed with breast cancer and 24.7% were gastrointestinal cancer. A majority presented with later-stage cancer, with 47.0% classified as Stage 4 and 32.3% as Stage 3. A significant portion of participants (78.1%) were undergoing chemotherapy treatment during the interview. Notably, about 39 study participants sought treatment at private facilities, although the costs associated with such facilities were excluded from the calculation. The majority of the participants (52.8%) had been living with a cancer diagnosis for less than a year. On average, the hospitalisation frequency was 1.68 (1.97) visits per year, with an average length of hospital stay of 10.29 (12.30) days, and an outpatient frequency of 30.11 (25.38) visits per year.

#### Out-of-pocket costs and productivity loss

Table 4 presents a detailed breakdown of the cost components and the overall costs of cancer. The median (IQR) and mean (SD) of medical costs were MYR 777.30 (MYR 1,648.25) and MYR 1,624.72 (MYR 2,593.63), while the non-medical costs were MYR 2,711.70 (MYR 3,398.60) and MYR 3,797.28 (MYR 3,788.04), respectively. The primary contributors to the average total OOP costs of MYR 5,422.00 (MYR 4,731.50) were transportation at MYR 1,707.94 (MYR 1,933.74), nutritional supplements at MYR 1,582.12 (MYR 2,862.41), outpatient treatment at MYR 761.39 (MYR 1,746.10), and medical supplies at MYR 601.09 (MYR 1,451.95).

**Table 4:** Out-of-pocket spending and productivity loss costs due to cancer.

Cost Components		Median (IQR)	Mean (SD)	% of total costs
**OOP**	**Medical**	**MYR 777.30 (1648.25)**	**MYR 1,624.72 (2593.63)**	**14.6**
	Inpatient treatment	0.00 (250.00)	262.24 (949.02)	
	Outpatient treatment	228.00 (885.00)	761.39 (1746.10)	
	Medical supplies^a^	0.00 (357.50)	601.09 (1451.95)	
	**Non-medical**	**MYR 2,711.70 (3398.60)**	**MYR 3,797.28 (3788.04)**	**34.2**
	Transportation^b^	1,146.00 (1725.60)	1,707.94 (1933.74)	
	Accommodation	0.00 (0.00)	103.12 (637.04)	
	Meal	120.00 (417.00)	324.61 (593.97)	
	Childcare	0.00 (0.00)	6.70 (117.98)	
	Nutritional supplements	676.00 (1843.20)	1,582.12 (2862.41)	
	Alternative treatment	0.00 (0.00)	72.79 (383.99)	
**Total OOP**	**Medical + Non-medical**	**MYR 4,112.00 (4,984.50)** **US$ 888.21 (1,076.68)**	**MYR 5,422.00 (4,731.50)** **US$ 1,171.18 (1,022.03)**	
**Productivity** **Loss**	**Paid and unpaid work**	**MYR 2,076.48 (8047.69)**	**MYR 5,680.85 (8375.17)**	**51.2**
	Absenteeism^c^	6,923.08 (21,923.08)	14,981.54 (16,653.84)	
	Limited productive activities^d^	1,846.08 (6,489.00)	4,593.75 (5,938.17)	
**Total Costs**	**OOP + Productivity Loss**	**MYR 8,254.32 (11,288.52)** **US$ 1,728.98 (2,438.39)**	**MYR 11,102.85 (10,040.19)** **US$ 2,398.28 (2,168.74)**	**100**

^a^ Medical supplies constituted of equipment and/or disposable items (e.g., breast prosthesis, stoma bag, diapers, needles, syringe, etc.)

^b^ Transportation includes fuel (mileage/km), toll, parking fees and public transport fees

^c^ Absent from work (employed, n = 49)

^d^ Inability to do routine household activities (unemployed, housewives, retired, n = 385)

Values are reported in MYR, and the final figures are presented in US$.

On the other hand, the median and mean productivity loss costs due to absenteeism were MYR 6,923.08 (MYR 21,923.08) and MYR 14,981.54 (MYR 16,653.84), respectively. Additionally, productivity loss due to limitations in productive activities incurred a median and mean costs of MYR 1,846.08 (MYR 6,489.00) and MYR 4,593.75 (MYR 5,938.17), respectively. In totality, these costs contribute to an average total costs per patient per year of approximately MYR 11,102.85 (MYR 10,040.19).

Highlighting the significance of these costs, productivity loss emerges as the major driver, constituting 51.2% of cancer patients’ total costs. Non-medical and medical costs contribute around 34.2% and 14.6% to the total costs, respectively.

While overall OOP costs for cancer patients are higher in Peninsular Malaysia than in Sabah and Sarawak, this difference is not statistically significant (S2 Appendix). However, a closer examination reveals that transportation costs, a significant component of total OOP expenses, differ markedly between these regions, with a significant *p*-value of 0.002. Table 5 provides a breakdown of the annual transportation costs aggregated by Peninsular Malaysia and Sabah and Sarawak. A significant difference (*p*<0.05) in the average round-trip travel distances was observed, with those in Peninsular Malaysia and Sabah and Sarawak covering 91.8 km and 261.5 km, respectively. Residents in the Peninsular spent an average of MYR 1,465.94 (MYR 1,429.20), while those in Sabah and Sarawak incurred a higher average expense of MYR 2,254.28 (MYR 2,502.05). Analysing the cost breakdown, participants from both Peninsular and Sabah and Sarawak allocated the highest expenditure to petrol, followed by public transport. Toll fees were covered exclusively by Peninsular residents, and parking fees were the lowest among the listed expenses.

**Table 5:** Annual transportation costs (MYR) breakdown.

	Peninsular (n = 298)		Sabah and Sarawak (n = 132)		*p*-value
	Median (IQR)	Mean (SD)	Median (IQR)	Mean (SD)	
Public transport	0.00 (0.00)	406.17 (1283.87)	0.00 (0.00)	793.89 (2,167.08)	0.183
Petrol	584.30 (1033.10)	845.09 (1027.55)	656.00 (1825.50)	1,418.73 (1,898.18)	0.041*
Toll fees	0.00 (209.90)	153.29 (269.11)	0.00 (0.00)	0.00 (0.00)	<0.001*
Parking fees	0.00 (80.00)	61.39 (137.24)	0.00 (0.00)	41.67 (102.84)	0.008*
**Total transport**	**MYR 1,040.00 (1,429.20)** **US$ 224.65** **(308.72)**	**MYR 1,465.94 (1,564.80)** **US$ 316.65** **(338.01)**	**MYR 1,540.00 (2,297.80)** **US$ 332.65** **(496.34)**	**MYR 2,254.28 (2,502.05)** **US$ 486.94 (540.46)**	**0.002** *

Values are reported in MYR, and the final figures are presented in US$.

*p<0.05 considered statistically significant using the Mann-Whitney U test.

### Factors associated with OOP expenditure

The final model for independent predictors for OOP costs due to cancer is presented in Table 6. Results of the multivariate regression analysis indicated that factors such as age, ethnicity, employment, monthly household income, and annual outpatient visits may influence OOP costs. Specifically, patients in the 50–59 age are experiencing less burden of paying OOP expenses (exp β = 0.74, 95% CI: 0.56–0.98; *p* = 0.03). Chinese ethnicity (exp β = 1.44, 95% CI: 1.15–1.79; *p*<0.001) showed higher OOP expenses during the cancer treatment compared to Malay ethnicity. Regarding employment status, retiree patients had lower OOP costs compared to those employed (exp β = 0.54; 95% CI: 0.37–0.79; *p* = 0.002). Additionally, patients in households with income MYR 2000–5000 and >5000 were associated with higher burdens of OOP costs (exp β = 1.31; 95% CI: 1.07–1.60; *p* = 0.01) and (exp β = 1.40; 95% CI: 1.10–1.78; *p* = 0.01), respectively. Furthermore, patients with higher annual outpatient visits (exp β = 1.00; 95% CI: 1.00–1.01; *p* = 0.02) were also linked to increased OOP expenses. The model demonstrated an adequate fit to the data, as indicated by the deviance of 0.91, Pearson Chi-Square statistic of 1.09, suggesting a reasonable explanation of the variance in OOP costs among the studied population. Additionally, the likelihood ratio test showed a significant improvement over the null model (*p* < 0.001), supporting the inclusion of the selected predictors.

**Table 6:** Factors affecting OOP costs on cancer patients using multivariate regression analysis.

Variables	Category	Exp β-coefficient	95% CI	*p*-value
Age (years)	40–49	Ref	Ref	Ref
	50–59	0.74	0.56–0.98	0.034*
	60–69	1.01	0.76–1.35	0.922
	≥70	1.00	0.71–1.41	0.991
Ethnicity	Malay	Ref	Ref	Ref
	Chinese	1.44	1.15–1.79	<0.001*
	Indian	0.84	0.62–1.15	0.275
	Indigenous Sabah	1.34	0.92–1.96	0.127
	Indigenous Sarawak	1.31	0.88–1.96	0.181
	Others	1.07	0.30–3.84	0.923
Marital Status	Married	Ref	Ref	Ref
	Divorced/ separated/ widow	0.86	0.67–1.45	0.264
	Retiree	1.04	0.75–1.12	0.818
Employment	Employed	Ref	Ref	Ref
	Unemployed	0.83	0.62–1.12	0.215
	Retiree	0.54	0.37–0.79	0.002*
Region	Peninsular Malaysia	Ref	Ref	Ref
	Sabah and Sarawak	0.79	0.60–1.05	0.107
Diagnosis (cancer site)	Breast	Ref	Ref	Ref
	Respiratory	0.98	0.72–1.34	0.910
	Gastrointestinal	1.25	0.98–1.59	0.069
	Urogenital	1.05	0.70–1.58	0.823
	Female reproductive	0.88	0.65–1.20	0.429
	Others	0.89	0.68–1.17	0.409
Present treatment	Chemotherapy	Ref	Ref	Ref
	Radiotherapy	1.23	0.95–1.59	0.116
	Surgery	1.29	0.72–2.32	0.389
	Chemotherapy + Radiotherapy	1.25	0.85–1.83	0.268
	Others	1.44	0.58–3.55	0.430
Time since diagnosis	<1 year	Ref	Ref	Ref
	≥1 year	0.85	0.71–1.02	0.075
Monthly household income (MYR)	<2000	Ref	Ref	Ref
	2000–4500	1.31	1.07–1.60	0.009*
	>4500	1.40	1.10–1.78	0.006*
^Ψ^Number of outpatient visits/ year	-	1.00	1.00–1.01	0.022*

^Ψ^Covariates = the continuous variables

^*^p<0.05 is considered statistically significant.

### Goddness-of-fit measures deviance of 0.914, Pearson Chi-Square of 1.094 and likelihood ratio Chi-Square of <0.001, indicates a well-fitting model

Univariate regression analysis (S3 Appendix) identified additional independent factors related to cancer OOP expenses, including age, ethnicity, marital status, employment, monthly household income, region, cancer diagnosis, time since diagnosis, present treatment and number of outpatient visits.

### Financial coping mechanisms

**Fig 2:**
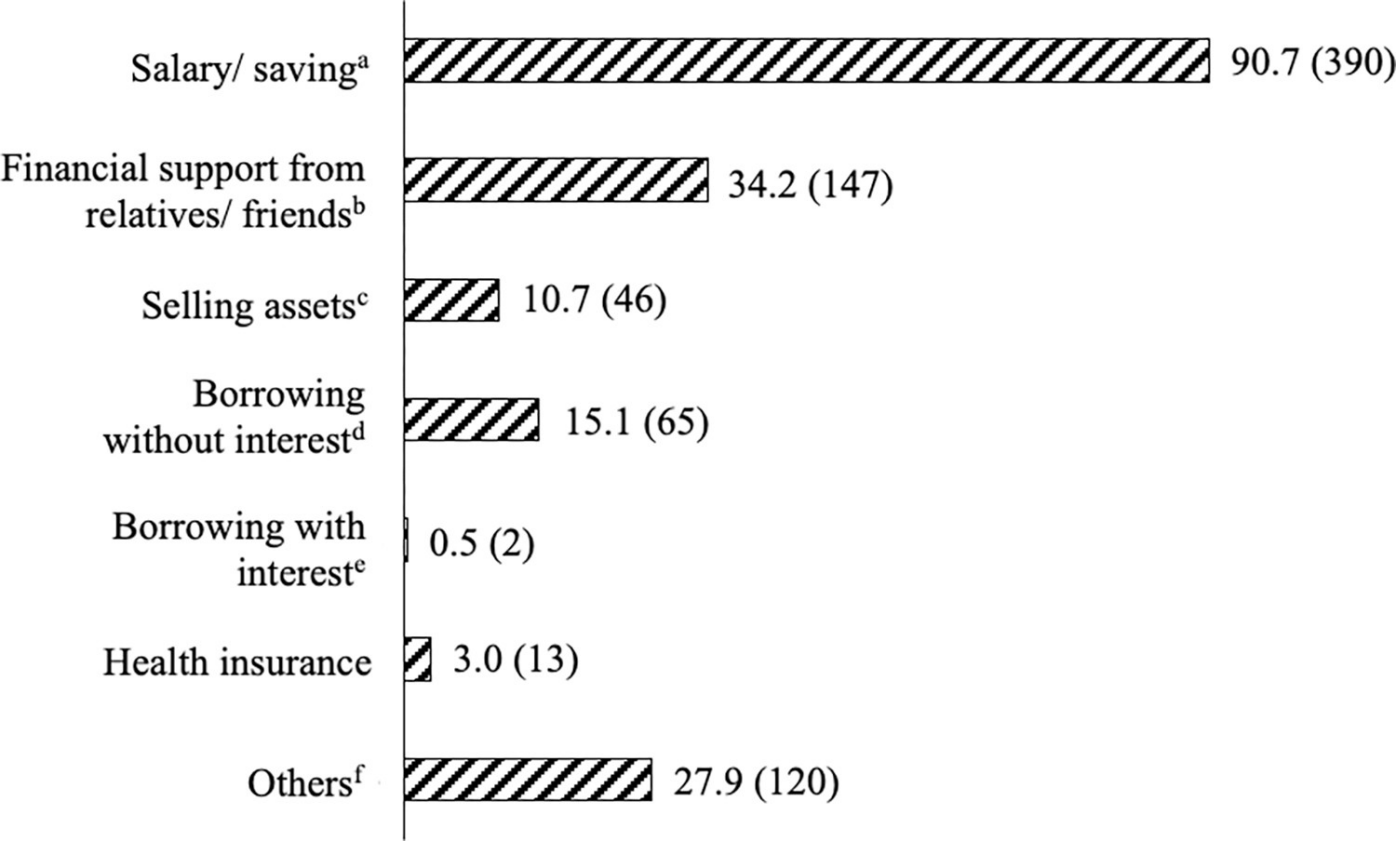
Financial coping strategies used by cancer patients to finance their cancer OOP expenses, % (n). Frequencies and percentage would not be added up because multiple responses were possible. ^a^ It includes salary and/ or savings (i.e., Employee’s Provident Fund, EPF) of personal and household; ^b^ Financial support received from relatives and friends which is non-refundable; ^c^ Any means of selling household assets (i.e., jewellery, property, land and other household items); ^d^ Borrowings or taking loans from individuals (non-household members or friends); ^e^ Borrowings or taking loans from financial institutions; ^f^ Other financial sources received from non-governmental organizations (i.e., MAKNA Cancer Support) and social welfare centres (i.e., Zakat).

The study participants adopted various coping strategies to address their cancer OOP expenses, as illustrated in Fig 2. A predominant majority (90.7%) relied on their own, besides household salary and savings, indicating a common approach to managing the financial burdens associated with cancer. About 34% of participants sought financial assistance from relatives and friends to alleviate the imposed financial strain. Furthermore, support from non-governmental organizations and social welfare centres played a role in covering expenses for 27.9% of participants. Additional funding was acquired through borrowing without interest (15.1%), the sale of assets (10.7%), utilisation of health insurance (3.0%), and the least common strategy of borrowing with interest (0.5%).

## Discussion

The study demonstrated that cancer places considerable financial strain on patients, even in a highly subsidized healthcare system such as Malaysia. Despite patients paying only a minimal fee at public healthcare facilities, their financial burden remained high. This was predominantly attributed to productivity loss and non-medical expenses. A shift in major cost contributors from medical to non-medical as often observed in other countries occurred because the subsidy extended only to medical treatment and hospital charges. This leaves the relative proportion of other additional expenses covered by the affected patients and households to be larger. Furthermore, the data also showed that even with subsidies, specific outpatient charges such as for radiotherapies and out-of-formulary medication purchases, in addition to medical aids can still add on to form a significant OOP medical expenditure. This was in line with several studies where such expenses can compound and in certain circumstances, become lifelong financial burdens for the patients [16,30–32].

Interestingly, travel and nutritional supplement costs surpass all other OOP cost categories. Transportation costs, frequently acknowledged as burdensome, appeared to hold significant importance, especially among individuals from lower socioeconomic statuses [16,30,32,33]. The combination of frequent visits and extended travel distances to cancer referral hospitals in this study contributed to the overall escalation of OOP costs. The high unit cost of transportation further magnifies the financial strain, particularly for cancer patients residing in distant locations. This is particularly true for the vast regions of Sabah and Sarawak, each with only one public cancer referral centre. The geographical limitations necessitate longer travel distances and higher expenses, including additional costs such as vehicle rentals, airplane tickets and boat fares, to reach cancer centres in urban areas. This limited access to cancer centres in these regions exacerbates the overall escalation of OOP costs. Additionally, expenditures were heightened by the intake of nutritional supplements, such as fortified milk and health supplements [16,30,34]. While the data was not able to distinguish whether such practices were prescribed or self-initiated, it is worth noting that such behaviours were also common among the lower-income groups.

The multivariate regression analysis identified as age, ethnicity, employment status, monthly household income, and the annual number of outpatient visits as significant predictors of OOP expenses among cancer patients. Contrary to some expectations, the analysis revealed that patients aged 50–59 experience a reduced burden of OOP expenses. This finding contrasts with a recent population-based study by Schneider et al. [35] among breast cancer patients in Germany, which highlighted increased OOP expenses among older patients. The difference in findings could be due to variations in healthcare utilization across age groups in different healthcare systems. Interestingly, while the mean costs calculated in this study indicate that patients aged 60 and above have higher spending, the GLM analysis suggests that the burden of OOP expenses is less for those aged 50–59. This discrepancy may be explained by differences in healthcare needs and financial capacities, where older individuals might face higher absolute costs due to more frequent healthcare utilization, but this does not necessarily translate to a higher relative burden after adjusting for other factors [36].

On the other hand, Chinese ethnicity was shown to also be a predictor for higher OOP expenditure. They specifically had a larger medical care expenditure compared to other ethnicities in public healthcare. Further analysis to explore possible reasons for such a trend showed that ethnicity was not associated with cancer types, severity, employment categories, and expenditures on nutritional supplements. The possible reason for this might align with a prior local study by Wan Puteh et al. [37] who postulated that the higher OOP burden among the minority groups may be associated with employment in the public sector. This is because in Malaysia charges are waived for current and retired civil servants. The current national statistics demonstrated that while ethnic minorities account for 42% of the population, only 22% are public service employees [38]. Thus, the additional fees waivered may have led to differences in the medical cost in the public sector.

Contrary to prior research which showed cancer patients residing in lower-income households were prone to higher OOP expenses [37,39], our data among the lower-income B40 households demonstrated that the poorest group with an income less than RM 2000 had a reduced likelihood of bearing a higher OOP burden. This discrepancy might be attributed to their scarce financial resources, which potentially limits their OOP expenditures, especially for non-medical expenses. While other studies have suggested that a longer time since diagnosis as well as higher hospitalisation frequency and longer length of hospital stay tend to influence OOP expenses [35,40], this study surprisingly reveals the opposite effect. Individuals with a higher number of outpatient visits, as supported by previous studies surprisingly bear a greater OOP burden [37,41].

The oversight of productivity loss estimation is evident in numerous cancer cost studies, despite its significant contribution to the overall costs of cancer care [6,42]. In reality, the costs attributed to productivity loss due to cancer could potentially constitute up to half of the overall expenditures [43]. This was echoed by the findings the average total productivity loss due to absenteeism and limitations in performing productive activities costs surpassed the OOP costs. The incapacity of cancer patients to work or the necessity for extended leaves for example not only affects their financial situations but also strains the overall income of the household. Moreover, the emotional toll and heightened caregiving responsibilities may further diminish the productivity of other household members, thereby creating a compounding effect on the economic well-being of the entire family [44,45]. It is worth highlighting that the losses are expected to be even higher if productivity loss from presenteeism and possible premature death were included in the long run.

Cancer patients may grapple with financial challenges even within a publicly funded healthcare system [46]. While patients can tap into their financial reserves, such as savings and health insurance, the persistent nature of cancer can swiftly deplete these resources. This study reveals a notable proportion of cancer patients experiencing unemployment, leaving them with little recourse other than relying on support from family members. However, in households lacking substantial initial savings, a cancer diagnosis can prove particularly devastating, resulting in financial sacrifices and household impoverishment that affect all members [47,48]. This study underscores that families with limited financial resources, impacted by cancer, often resort to borrowing and selling assets to meet associated costs. This finding aligns with previous studies that highlighted selling assets and borrowings as major financial coping strategies [49–52]. Moreover, a study by Azzani et al. [4] reported that households of colorectal cancer patients resort to selling household items or borrowing money from relatives and friends to navigate their new financial circumstances. Despite the presence of subsidies in public healthcare facilities, patients still find themselves reaching into their own pockets to cover non-subsidized treatments, medications, and hidden non-medical costs.

It was also observed that patients and their family members allocated nearly 34% of their total monthly income to finance cancer OOP expenses. This percentage greatly contrasts with the Malaysian household expenditure on healthcare, which usually ranges from 2.6% to 3.5% of the total monthly income [53]. Essentially, the costs associated with cancer impose a noticeable economic strain on those affected. A study in Canada reported that among cancer patients who perceive OOP as significant spent, on average, 34% of their total monthly income [54]. Similarly, in Southeastern United States, breast cancer survivors allocated as much as 31% of their monthly income to these expenses [39]. Given that Malaysia’s healthcare expenditure is currently below the WHO-recommended threshold (< 5%–6% of the annual GDP), allocating additional funds for healthcare, subsidising the costs of medical aid, and providing financial assistance programme could potentially alleviate the financial burden and improve healthcare access among lower-income cancer patients [55,56].

This study is subject to several limitations. Firstly, the estimation of expenses is confined to public referral cancer centres under the MOH. Thus, possible expenses incurred at private hospitals were not included. Eventhough evidence showed that lower-income patients are less likely to pursue care in private facilities [57], our sample showed that patients may still seek selected services such as surgery and investigations in private facilities. Thus, it may lead to an underestimation of the total financial impact on patients. Future research should consider including both public and private sector costs to provide a more comprehensive assessment. Secondly, the reported OOP spending and productivity loss are also limited by the recall period. This approach could result in cost exaggeration or underestimation, depending on the type of service and the length of the recall period, leading to potential inconsistencies in the data. However, a pilot study was conducted prior to the main study to evaluate these recall periods, and it was found that patients were generally able to recall and provide the required information accurately, supporting the feasibility of this approach despite the limitations. Furthermore, the study did not include the loss of productivity of caregivers due to the unavailability of companions for most interviews. This gap in knowledge, in addition to inaccurate recall of absenteeism, tends to grossly underestimate productivity losses. Lastly, the annualization of costs using one and three months’ expenses may potentially have influenced the accuracy of the total costs of cancer calculated in this study. This is because cancer expenditures are dependent on time, with expenses peaking in the first six months of diagnosis and year.

Albeit limitations raised, this study supplements the expanding body of literature addressing the financial burden of cancer from the patient’s perspective. Acknowledging the significant financial strain accompanying a cancer diagnosis is crucial for developing a patient-centred plan that addresses the specific needs of both patients and their families. The findings from this study play a pivotal role in advancing initiatives aimed at addressing the financial hardships experienced by low-income cancer patients [58], providing an estimate of the OOP expenses related to cancer care. Furthermore, the study highlights the ongoing role of household members as primary contributors to funding cancer-related care, even within publicly subsidised healthcare systems. Based on these findings, the PeKa B40 scheme, which offers financial assistance for cancer treatment and transportation appears to be timely and well-needed. This initiative commendably alleviates the financial burden on cancer patients, especially for expenses such as transportation and medical equipment [58]. However, it is worth exploring whether these initiatives reach the lower-income groups and if they alleviate their financial burdens. This study is thus poised to facilitate discussions around planning and decision-making on the redistribution of allocations based on needs and risks.

## Conclusions

In summary, this research emphasizes the significant impact of cancer OOP expenditures on the financial well-being and productivity of lower-income households. These families grapple with the dual challenges of reduced income and increased expenses. Despite the presence of heavily subsidized public healthcare as the foundation of universal health coverage in Malaysia, it may remain inadequate to shield cancer patients and their families from catastrophic expenditures. Thus, there is still a need for policymakers to incorporate a social safety net into the healthcare financing system, with a focus on prioritizing the disadvantaged population. Such integration should ensure access to health services without subjecting individuals to excessive financial strain while balancing the long-term sustainability of public healthcare. The implementation of targeted measures such as PeKa B40 cancer incentives [59] may greatly benefit these population subgroups. Furthermore, the inclusion of health insurance coverage and financial support schemes also has the potential to alleviate the financial difficulties faced by affected patients. Ultimately, it is hoped that the findings will help guide policy-makers in developing holistic patient-centred cancer care while planning for future resource reallocations and investments.

## Supporting information

S1 Appendix Study questionnaires. (PDF)

S2 Appendix Distribution of total OOP cancer costs by sociodemographic profiles. All costs are reported in MYR; *Mann-Whitney U test; **Kruskal-Wallis test.
(PDF)

S3 Appendix Predictor variables related to OOP costs due to cancer using univariate regression analysis. ^Ψ^Covariates = the continuous variables; *Variables with *p*<0.25 were fitted into the final model.
(PDF)

## References

[1] Zafar SY, Peppercorn JM, Schrag D, Taylor DH, Goetzinger AM, Zhong X, et al. The financial toxicity of cancer treatment: A pilot study assessing out-of-pocket expenses and the insured cancer patient’s experience. Oncologist [Internet]. 2013;18(4):381–90. Available from: doi: 10.1634/theoncologist.2012-0279 23442307 PMC3639525

[2] Guirguis S, Fitch M, Maganti M, Gupta AA, D’Agostino N, Korenblum C, et al. Biopsychosocial factors associated with supportive care needs in Canadian adolescent and young adult cancer survivors. J Clin Med [Internet]. 2021;10(12):2628. Available from: doi: 10.3390/jcm10122628 34203795 PMC8232806

[3] Angioli R, Capriglione S, Aloisi A, Miranda A, de Cicco Nardone C, Terranova C, et al. Economic impact among family caregivers of patients with advanced ovarian cancer. Int J Gynecol Cancer [Internet]. 2015;25(8):1541–6. Available from: doi: 10.1097/IGC.0000000000000512 26270119

[4] Azzani M, Roslani AC, Su TT. Financial burden of colorectal cancer treatment among patients and their families in a middle-income country. Support Care Cancer [Internet]. 2016;24(10):4423–32. Available from: doi: 10.1007/s00520-016-3283-2 27225528

[5] McCormick PJ. Cancer tsunami: Emerging trends, economic burden, and perioperative implications. Curr Anesthesiol Rep [Internet]. 2018;8(4):348–54. Available from: doi: 10.1007/s40140-018-0294-1 31130826 PMC6530937

[6] Longo CJ, Fitch M, Deber RB, Williams AP. Financial and family burden associated with cancer treatment in Ontario, Canada. Support Care Cancer [Internet]. 2006;14(11):1077–85. Available from: doi: 10.1007/s00520-006-0088-8 16896878

[7] The ACTION Study Group. Catastrophic health expenditure and 12-month mortality associated with cancer in Southeast Asia: results from a longitudinal study in eight countries. BMC Med [Internet]. 2015;13(1). Available from: 10.1186/s12916-015-0433-1. 26282128 PMC4539728

[8] Guy GP Jr, Yabroff KR, Ekwueme DU, Virgo KS, Han X, Banegas MP, et al. Healthcare expenditure burden among non-elderly cancer survivors, 2008–2012. Am J Prev Med [Internet]. 2015;49(6):S489–97. Available from: doi: 10.1016/j.amepre.2015.09.002 26590644 PMC6051701

[9] Fenn K. Is the financial burden of cancer impacting survivors’ quality of life? [Internet]. 2014 [cited 2024 Aug 12]. Available from: https://elischolar.library.yale.edu/ymtdl/1875.

[10] Callander E, Bates N, Lindsay D, Larkins S, Topp SM, Cunningham J, et al. Long-term out of pocket expenditure of people with cancer: comparing health service cost and use for indigenous and non-indigenous people with cancer in Australia. Int J Equity Health [Internet]. 2019;18(1). Available from: doi: 10.1186/s12939-019-0931-4 30755217 PMC6371603

[11] Kim W, Han K-T, Kim S. Do patients residing in provincial areas transport and spend more on cancer treatment in Korea? Int J Environ Res Public Health [Internet]. 2021;18(17):9247. Available from: doi: 10.3390/ijerph18179247 34501835 PMC8431159

[12] Mahmud A, Aljunid SM. Availability and accessibility of subsidized mammogram screening program in peninsular Malaysia: A preliminary study using travel impedance approach. PLoS One [Internet]. 2018;13(2):e0191764. Available from: 10.1371/journal.pone.0191764. PMC5794099 29389972

[13] Zahnd WE, Davis MM, Rotter JS, Vanderpool RC, Perry CK, Shannon J, et al. Rural-urban differences in financial burden among cancer survivors: an analysis of a nationally representative survey. Support Care Cancer [Internet]. 2019;27(12):4779–86. Available from: doi: 10.1007/s00520-019-04742-z 30972645 PMC6786922

[14] Yahya N, Sukiman NK, Suhaimi NA, Azmi NA, Manan HA. How many roads must a Malaysian walk down? Mapping the accessibility of radiotherapy facilities in Malaysia. PLoS One [Internet]. 2019;14(3):e0213583. Available from: 10.1371/journal.pone.0213583. PMC6428267 30897166

[15] Sledge GW. Patients and physicians in the era of modern cancer care. JAMA [Internet]. 2019;321(9):829. Available from: doi: 10.1001/jama.2018.17334 30768154

[16] Aminuddin F, Bahari MS, Zainuddin NA, Mohd Hanafiah AN, Mohd Hassan NZA. The direct and indirect costs of cancer among the lower-income group: Estimates from a pilot and feasibility study. Asian Pac J Cancer Prev [Internet]. 2023;24(2):489–96. Available from: doi: 10.31557/APJCP.2023.24.2.489 36853297 PMC10162611

[17] Department of Statistics Malaysia. Household Expenditure Survey Report, Malaysia 2022 [Internet]. 2023, July. Available from: https://storage.googleapis.com/dosm-public-publications/multiyear_2022_hies.pdf.

[18] Azizah AM, Hashimah B, Nirmal K., Siti Zubaidah AR, Puteri NA, Nabihah A, et al. Malaysia National Cancer Registry Report (MNCR) 2012–2016 [Internet]. 2019, June. Available from: https://www.moh.gov.my/moh/resources/Penerbitan/Laporan/Umum/2012-2016%20(MNCRR)/MNCR_2012-2016_FINAL_(PUBLISHED_2019).pdf.

[19] Koris R, Mohamed Nor N, Haron S, Hamid T, Muhammad Nur A, Ismail N, et al. The cost of healthcare among Malaysian community-dwelling elderly. J Ekon Malays [Internet]. 2019;53(1). Available from: 10.17576/jem-2019-5301-8.

[20] Choong C, Mohamed Firouz AM, Jasmin AF, Muhammad Noor N, Gong R. Time to care: Gender inequality, unpaid care work and time use survey [Internet]. Khazanah Research Institute; 2019. Available from: https://www.krinstitute.org/assets/contentMS/img/template/editor/Publications_Time%20to%20Care_Full%20report.pdf.

[21] Universiti Putra Malaysia. Retirement Preparedness and Productive Ageing among Government Employees and Retirees in Klang Valley (Final Report) [Internet]. 2018. Available from: https://www.kwap.gov.my/documents/publications/others/Myageing_report.pdf.

[22] John D, Kumar V. Exposure to hardship financing for healthcare among rural poor in Chhattisgarh, India. J Health Manag [Internet]. 2017;19(3):387–400. Available from: 10.1177/0972063417717879.

[23] Mohd Hassan NZA, Mohd Nor Sham Kunusagaran MSJ, Zaimi NA, Aminuddin F, Ab Rahim FI, Jawahir S, et al. The inequalities and determinants of Households’ Distress Financing on Out-off-Pocket Health expenditure in Malaysia. BMC Public Health [Internet]. 2022;22(1). Available from: doi: 10.1186/s12889-022-12834-5 35255884 PMC8900333

[24] Yadav J, John D, Menon G. Out of pocket expenditure on tuberculosis in India: Do households face hardship financing? Indian J Tuberc [Internet]. 2019;66(4):448–60. Available from: doi: 10.1016/j.ijtb.2019.02.016 31813431

[25] Lee SM, Choi IS, Han E, Suh D, Shin EK, Je S, et al. Incremental treatment costs attributable to overweight and obesity in patients with diabetes: Quantile regression approach. Obesity (Silver Spring) [Internet]. 2018;26(1):223–32. Available from: doi: 10.1002/oby.22080 29178436

[26] Dodd S, Bassi A, Bodger K, Williamson P. A comparison of multivariable regression models to analyse cost data. J Eval Clin Pract [Internet]. 2006;12(1):76–86. Available from: doi: 10.1111/j.1365-2753.2006.00610.x 16422782

[27] Manning WG, Mullahy J. Estimating log models: to transform or not to transform? J Health Econ [Internet]. 2001;20(4):461–94. Available from: doi: 10.1016/s0167-6296(01)00086-8 11469231

[28] Mihaylova B, Briggs A, O’Hagan A, Thompson SG. Review of statistical methods for analysing healthcare resources and costs. Health Econ [Internet]. 2011;20(8):897–916. Available from: doi: 10.1002/hec.1653 20799344 PMC3470917

[29] Bank Negara Malaysia. [cited 2023 Spring 8]. Available from: https://www.bnm.gov.my/exchange-rates?p_p_id=bnm_exchange_rate_display_portlet&p_p_lifecycle=0&p_p_state=normal&p_p_mode=view&_bnm_exchange_rate_display_portlet_monthStart=7&_bnm_exchange_rate_display_portlet_yearStart=2023&_bnm_exchange_rate_display_portlet_monthEnd=7&_bnm_exchange_rate_display_portlet_yearEnd=2023&_bnm_exchange_rate_display_portlet_sessionTime=1700&_bnm_exchange_rate_display_portlet_rateType=MR&_bnm_exchange_rate_display_portlet_quotation=rm.

[30] Fnu N, Kuan WC, Kong YC, Bustamam RS, Wong LP, Subramaniam S, et al. Cancer-related costs, the resulting financial impact and coping strategies among cancer survivors living in a setting with a pluralistic health system: a qualitative study. Ecancermedicalscience [Internet]. 2022;16. Available from: 10.3332/ecancer.2022.1449. PMC9666287 36405936

[31] Hanly P, Maguire R, Ceilleachair AO, Sharp L. Financial hardship associated with colorectal cancer survivorship: The role of asset depletion and debt accumulation. Psychooncology [Internet]. 2018;27(9):2165–71. Available from: doi: 10.1002/pon.4786 29852528

[32] Kong YC, Wong LP, Ng CW, Taib NA, Bhoo-Pathy NT, Yusof MM, et al. Understanding the financial needs following diagnosis of breast cancer in a setting with universal health coverage. Oncologist [Internet]. 2020;25(6):497–504. Available from: doi: 10.1634/theoncologist.2019-0426 31922332 PMC7288648

[33] Raman S, Shafie A, Abraham MT, Shim CK, Maling TH, Rajendran S, et al. Household catastrophic health expenditure from oral potentially malignant disorders and oral cancer in public healthcare of Malaysia. Asian Pac J Cancer Prev [Internet]. 2022;23(5):1611–8. Available from: doi: 10.31557/APJCP.2022.23.5.1611 35633545 PMC9587868

[34] Yen SH, Shatar AK, Hashim H. Exploring the economic impact on breast cancer patients in Kelantan. Perspektif Jurnal Sains Sosial dan Kemanusiaan. 2015;7(2):43–56.

[35] Schneider J, Hernandez D, CAESAR study group, Schlander M, Arndt V, on behalf of the CEASAR study group. Out-of-pocket payments and loss of income among long-term breast cancer survivors in Germany: a multi-regional population-based study. J Cancer Surviv [Internet]. 2023;17(6):1639–59. Available from: doi: 10.1007/s11764-022-01293-x 36459378 PMC10539192

[36] Newton JC, Johnson CE, Hohnen H, Bulsara M, Ives A, McKiernan S, et al. Out-of-pocket expenses experienced by rural Western Australians diagnosed with cancer. Support Care Cancer [Internet]. 2018;26(10):3543–52. Available from: doi: 10.1007/s00520-018-4205-2 29704109

[37] Wan Puteh SE, Abdullah YR, Aizuddin AN. Catastrophic health expenditure (CHE) among cancer population in a middle income country with universal healthcare financing. Asian Pac J Cancer Prev [Internet]. 2023;24(6):1897–904. Available from: 10.31557/apjcp.2023.24.6.1897. 37378917 PMC10505870

[38] Aun LH. Diversity in Malaysia’s civil service: From venting old grouses to seeking new grounds [Internet]. Edu.sg. [cited 2024 Aug 12]. Available from: https://www.iseas.edu.sg/wp-content/uploads/2023/03/ISEAS_Perspective_2023_34.pdf.

[39] Pisu M, Azuero A, Benz R, McNees P, Meneses K. Out‐of‐pocket costs and burden among rural breast cancer survivors. Cancer Med [Internet]. 2017;6(3):572–81. Available from: doi: 10.1002/cam4.1017 28229562 PMC5345680

[40] Xia Y, Chen Y, Chen J, Gan Y, Su C, Zhang H, et al. Measuring direct non-medical burden among patients with advanced non-small cell lung cancer in China: is there a difference in health status? Front Public Health [Internet]. 2023;11. Available from: 10.3389/fpubh.2023.1090623. PMC10192575 37213608

[41] Sui M, Zeng X, Tan WJ, Tao S, Liu R, Liu B, et al. Catastrophic health expenditures of households living with pediatric leukemia in China. Cancer Med [Internet]. 2020;9(18):6802–12. Available from: doi: 10.1002/cam4.3317 32697427 PMC7520357

[42] Jonnson B, Wilking N. The burden and cost of cancer. Annals of Oncology. 2007;18:8–22.

[43] Singleterry J, American Cancer Society Cancer Action Network. The costs of cancer [Internet]. 2017 [cited 2023 Aug 9]. Available from: https://www.fightcancer.org/sites/default/files/Costs%20of%20Cancer%20-%20Final%20Web.pdf.

[44] Ferrell BR, Kravitz K. Cancer Care: Supporting underserved and financially burdened family caregivers. J Adv Pract Oncol. 2017;8(5):494–500. 30079266 PMC6067909

[45] Howard AF, Lynch K, Thorne S, Porcino A, Lambert L, De Vera MA, et al. Occupational and financial setbacks in caregivers of people with colorectal cancer: Considerations for caregiver-reported outcomes. Curr Oncol [Internet]. 2022;29(11):8180–96. Available from: doi: 10.3390/curroncol29110646 36354706 PMC9689650

[46] Parker C, Berkovic D, Wei A, Zomer E, Liew D, Ayton D. ‘If I don’t work, I don’t get paid’: An Australian qualitative exploration of the financial impacts of acute myeloid leukaemia. Health Soc Care Community [Internet]. 2022;30(5). Available from: 10.1111/hsc.13642. 34766671

[47] Banegas MP, Schneider JL, Firemark AJ, Dickerson JF, Kent EE, de Moor JS, et al. The social and economic toll of cancer survivorship: a complex web of financial sacrifice. J Cancer Surviv [Internet]. 2019;13(3):406–17. Available from: doi: 10.1007/s11764-019-00761-1 31123985 PMC6724195

[48] Fu W, Shi J, Zhang X, Liu C, Sun C, Du Y, et al. Effects of cancer treatment on household impoverishment: a multicentre cross-sectional study in China. BMJ Open [Internet]. 2021;11(6):e044322. Available from: doi: 10.1136/bmjopen-2020-044322 34193481 PMC8246348

[49] Bogale T, Mariam DH, Ali A. Costs of illness and coping strategies in a coffee-growing rural district of Ethiopia. J Health Popul Nutr. 2005;23(2):192–9. 16117372

[50] Kruk ME, Goldmann E, Galea S. Borrowing and selling to pay for health care in low- and middle-income countries. Health Aff (Millwood) [Internet]. 2009;28(4):1056–66. Available from: doi: 10.1377/hlthaff.28.4.1056 19597204

[51] Leive A. Coping with out-of-pocket health payments: empirical evidence from 15 African countries. Bull World Health Organ [Internet]. 2008;86(11):849–56. Available from: doi: 10.2471/blt.07.049403 19030690 PMC2649544

[52] Pourreza A, Harirchi I, Bazyar M. Differentiation of out-of-pocket expenditures in cancer patients; a case study in the cancer institute of Iran. Evid Based Health Policy Manage Econ. 2017;1:65–73.

[53] Abdullah Yusof S, Duasa J. Household decision-making and expenditure patterns of married men and women in Malaysia. J Fam Econ Issues [Internet]. 2010;31(3):371–81. Available from: 10.1007/s10834-010-9200-9.

[54] Longo CJ, Fitch MI, Loree JM, Carlson LE, Turner D, Cheung WY, et al. Patient and family financial burden associated with cancer treatment in Canada: a national study. Support Care Cancer [Internet]. 2021;29(6):3377–86. Available from: doi: 10.1007/s00520-020-05907-x 33403399 PMC8062343

[55] Adams A, Kluender R, Mahoney N, Wang J, Wong F, Yin W. The impact of financial assistance programs on health care utilization: Evidence from Kaiser Permanente. Am Econ Rev Insights [Internet]. 2022;4(3):389–407. Available from: doi: 10.1257/aeri.20210515 36338144 PMC9634821

[56] Graham WCK, Bilger M. Financing long‐term services and supports: Ideas from Singapore. Milbank Q [Internet]. 2017;95(2):358–407. Available from: doi: 10.1111/1468-0009.12264 28589606 PMC5461396

[57] Khazanah Research Institute. Social Inequalities and Health in Malaysia: The State of Households 2020 Part III. Khazanah Research Institute [Internet]. 2020. Available from: https://www.krinstitute.org/assets/contentMS/img/template/editor/KRI%20-%20Full%20Report%20-%20Social%20Inequalities%20and%20Health%20in%20Malaysia_latest.pdf.

[58] Tucker-Seeley RD, Yabroff KR. Minimizing the “financial toxicity” associated with cancer care: advancing the research agenda. J Natl Cancer Inst [Internet]. 2016;108(5):djv410. Available from: doi: 10.1093/jnci/djv410 26657336

[59] Abdullah N, Rozali ZI, Gan SC, Ismail NA, Mohd Hazman NH, Abdul Jabar M, et al. PeKa B40 Report 2019–2020 [Internet]. 2021. Available from: https://protecthealth.com.my/wp-content/uploads/2021/10/PeKaB40_Report_2019-2020.pdf.

