# Supplementary material for: Cancer impact on lower-income patients in Malaysian public healthcare: An exploration of out-of-pocket expenses, productivity loss, and financial coping strategies
Source: PLoS One. 2024 Oct 9;19(10):e0311815. doi: 10.1371/journal.pone.0311815 (PMC11463769; doi:10.1371/journal.pone.0311815)
Supplement: S1 Appendix — (PDF) [file pone.0311815.s001.pdf]

BORANG SOAL SELIDIK/ QUESTIONNAIRE

ISI KANDUNGAN / *CONTENT*		
Seksyen/ Section	Kandungan / Contents	Muka Surat / Pages
A	Sociodemografi<br>*Sociodemography*	2-3
B	Kos perubatan langsung<br>*Direct medical cost*	4-5
C	Kos bukan perubatan langsung<br>*Direct non-medical cost*	6-8
D	Kos tidak langsung (kehilangan produktiviti)<br>*Indirect cost (Productivity loss)*	9-10
E	Perbelanjaan isi rumah<br>*Household expenditure*	11

TAJUK KAJIAN/ PROJECT TITLE:

THE FINANCIAL BURDEN OF CANCER: ESTIMATES FROM PATIENTS AMONG THE PeKa B40 BENEFICIARIES UNDERGOING CANCER TREATMENT IN SELECTED HOSPITALS (DICC-B40)

PENYELIDIK UTAMA/ PRIMARY INVESTIGATOR:

DR. FARHANA AMINUDDIN

Study ID: __________

**A. Sociodemografi/ *Sociodemographic***	
1. Tarikh pada hari anda menjawab soal selidik ini<br>*The date you are filling this questionnaire*<br>day ☐☐ month ☐☐ year ☐☐☐☐	
2. Adakah anda penerima 'Bantuan Sara Hidup' (BSH)?<br>*Are you a recipient of life assistance?*	☐ Ya/ *Yes*<br>☐ Tidak/ *No*
3. Jantina<br>*Gender*	☐ Lelaki/ *Male*<br>☐ Perempuan/ *Female*
4. Etnik<br>*Ethnicity*	☐ Melayu/ *Malay*<br>☐ Cina/ *Chinese*<br>☐ India/ *Indian*<br>☐ Bumiputera Sabah/ *Indigenous Sabah*<br>☐ Bumiputera Sarawak/ *Indigenous Sarawak*<br>☐ Lain-lain/ *Others*
5. Tahap Pengajian<br>*Education Level*	☐ Tidak bersekolah/ *No formal Education*<br>☐ Sekolah Rendah/ *Middle school*<br>☐ Sekolah Menengah/ *High school*<br>☐ Diploma/ *Diploma*<br>☐ Sarjana Muda/ *Bachelor's Degree*<br>☐ Sarjana atau PhD/ *Master's or PhD*
6. Status Perkahwinan<br>*Marital status*	☐ Berkahwin/ *Married*<br>☐ Bujang/ *Not married*<br>☐ Bercerai/ Berpisah/ Janda/<br>*Divorced/ Separated/Widow*
7. Status Pekerjaan<br>*Employment status*	☐ Kerajaan/ *Government or public sector*<br>☐ Swasta/ *Private sector*<br>☐ Bekerja Sendiri/ *Self employed*<br>☐ Tidak Bekerja/ *Unemployed*<br>☐ Pesara/ *Pensioner*<br>☐ Lain-lain/ *Others,*<br>Sila nyatakan/ *Please specify:*<br>……………………………………………

8. Perubahan terhadap Status Bekerja<br>*Change of employment status*<br>*(conditional on employed at diagnosis)*	☐ Tiada Perubahan/ *No change*<br>☐ Berhenti Kerja/ *Completely stop working*<br>☐ Cuti Sakit/ *Extended leave*<br>☐ Lain-lain/ *Others:*<br>Sila nyatakan/ *Please specify:*<br><br>………………………………………
9. Pendapatan kasar **bulanan** anda daripada bekerja atau pencen<br>*Your **monthly** gross income from work or pension*	i. Gaji atau Pencen/ *Salary or Pension*<br><br>RM……………………………..<br>ii. Sumbangan dari ahli keluarga (tinggal berasingan)/ *Donations from family members (living separately)*<br><br>RM……………………………..<br>iii. Lain-lain (sumbangan MAKNA, zakat, dll)/ *Others (donations from MAKNA, zakat, etc.)*<br><br>RM……………………………..
10. Berapakah bilangan ahli isi rumah anda?<br>*How many members do you have in your household?*	………………………………orang/ *people*
11. Pendapatan kasar bulanan seluruh ahli isi rumah<br>*Monthly gross income of all household members*	RM……………………………..

B. Kos Perubatan Langsung/ Direct Medical Cost

Soalan berikutnya adalah berkaitan dengan kos perubatan untuk rawatan kanser anda tempoh **3 bulan yang lepas**
*The following questions refer to the medical costs for your cancer treatment for the **past 3 months***

1. Apakah jenis kanser yang anda hidapi?
   What type of cancer are you suffering from?

   Jenis kanser/ *Cancer type* : ....
   Tahap kanser/ *Cancer stage* : ....

2. Bilakah anda didiagnosa sebagai pesakit kanser?
   When were you diagnosed with cancer?
   Hari/day bulan/month tahun/year
   ☐☐ ☐☐ ☐☐☐☐

3. Adakah anda dimasukkan ke wad (rawatan dalam) dalam tempoh **1 tahun** yang lepas? Jika Ya, sila nyatakan:
   *Have you been admitted to the ward (inpatient) in the last **1 year**? If Yes, please specify:*

Jumlah lawatan/ *How many times*	Jumlah hari/ *How many days*	Tujuan/ *Reason*	Bayaran (RM)/ *Charges (RM)*

4. Adakah anda ke hospital dalam tempoh **3 bulan** yang lepas untuk mendapatkan rawatan susulan, farmasi, rawatan harian dll. (selain kemasukan ke wad).
   *Have you been to the hospital in the last **3 months** for follow-up, daycare services, pharmacy, etc. (except admitted to the ward)*

Jumlah lawatan/ *How many times*	Tujuan/ *Reason*	Bayaran (RM)/ *Charges (RM)*

5. Adakah anda berbelanja untuk **peralatan perubatan** bagi rawatan kanser ini (e.g. prosthesis payudara, kerusi roda, beg stoma)? Jika Ya, sila nyatakan:
*Do you spend on **medical items** for this cancer treatment (e.g. breast prosthesis, wheelchair, stoma bag)? If Yes, please specify:*

Peralatan perubatan/ Medical equipment	Bayaran (RM)/ *Charges (RM)*

6. Adakah anda mendapat bayaran balik atau tuntutan semula?
Did you get reimbursement or refund?
☐ Tidak/ *No*
☐ Ya/ *Yes*

Jika Ya, berapakah bayaran yang dikembalikan kepada anda?
If yes, how much is the refund?

RM…………………………………………

7. Adakah anda menerima bantuan untuk rawatan kanser? Jika Ya, apakah jenis bantuan yang diterima?
Do you receive assistance (financial or medical items for the cancer treatment? If Yes, please specify:

Jenis bantuan *Type of assistance*	Organisasi *Organisation*	Bayaran (RM)/ *Charges (RM)*
Kewangan *Financial*		
Peralatan perubatan *Medical items*		

Contoh organisasi termasuk MAKNA, Zakat, JKM dan lain-lain.
Organisations such as MAKNA, Zakat, JKM etc.

C. Kos Bukan Perubatan Langsung/ *Direct Non-Medical Cost*

Soalan berikutnya adalah berkaitan dengan kos yang dikeluarkan oleh anda untuk mendapatkan rawatan kanser bagi tempoh **3 bulan yang lepas**
The following questions refer to your direct non-medical cost in receiving cancer treatment for the ***past 3 months***

1. Bagaimanakah cara anda pergi ke hospital?
   How do you go to the hospital?

   ☐ Kenderaan sendiri/ *Own transport*
   ☐ Pengangkutan awam/ *Public transport*
   ☐ Pertolongan dari ahli keluarga atau jiran/ *Help from family members or neighbour*
   ☐ Lain-lain, sila nyatakan/ *Other, please specify* ……………………………………

2. Berapakah jarak perjalanan anda dari rumah ke hospital?
   What is the distance from your home to the hospital?

   ☐ 5 – 10 km / *5 – 10 km*
   ☐ 11- 40 km/ *11- 40 km*
   ☐ 41-70 km/ *41- 70 km*
   ☐ >70 km/ *>70 km*…

   OR Please specify: ……………………….

3. Semasa anda mendapatkan rawatan, berapa anda belanja untuk:
   In the process of obtaining treatment, how much do you spend on:

   Jika tidak berkaitan, sila isi '0'
   Please answer '0' if not applicable

a. Pengangkutan, contohnya kereta sewa, tambang teksi/ Grab car/ bas<br>*Transportation, for example, rental car, fees for taxi/ Grab car/ bus*	RM…………………………
b. Jika menggunakan kenderaan sendiri:<br>B1: Petrol/ *Fuel*<br>B2: Tol/ *Toll fares*<br>B3: Bayaran meletak kenderaan/ *Parking fee*	RM…………………………<br>RM…………………………<br>RM…………………………
c. Tempat untuk menginap, seperti rumah tumpangan, hotel dan lain-lain<br>*Places to stay, such as guesthouse, hotel and others*	RM…………………………
d. Makanan<br>*Meal*	RM…………………………

e. Penjagaan anak<br>*Childcare*	RM…………………………
f. Others<br>*Lain-lain:*	RM…………………………

4. Adakah anda mendapatkan vitamin dan suplemen seperti kalsium, minyak ikan, herba, spirulina, "evening primrose oil" dll.? (Untuk tempoh 3 bulan yang lepas)
Do you get vitamins and supplements such as calcium, fish oil, herbal, spirulina, evening primrose oil, etc.) (For the last 3 months)

RM …………………

Adakah makanan tambahan ini disyorkan atau ditetapkan oleh pengamal perubatan?
Are the supplemental foods recommended or prescribed by the clinician?
☐ Tidak/ *No*
☐ Ya/ *Yes*

5. Adakah anda mendapatkan rawatan alternatif, contohnya rawatan tradisional? Jika ya, berapakah perbelanjaan anda untuk setiap rawatan? (Untuk tempoh 3 bulan yang lepas)
Do you seek for alternative treatment, for example traditional treatment? If yes, how much did you spend each time you receive the treatment? (For the last 3 months)

RM …………………

6. Dalam tempoh 12 bulan lepas, dari mana isirumah anda mendapatkan wang untuk membayar perbelanjaan kesihatan anda?
In the last 12 months, where did your household get the money to pay for your health expenses?

a. Wang simpanan<br>*Savings*	☐ Ya/ *Yes*<br>☐ Tidak/ *No*
b. Bayaran atau tuntutan daripada insurans kesihatan<br>*Payment or reimbursement from health insurance*	☐ Ya/ *Yes*<br>☐ Tidak/ *No*
c. Jual/ gadai barang-barang seperti perabot, haiwan, barang kemas, dan lain-lain<br>*Sold/ pawned items such as furniture, animals, jewellery and others*	☐ Ya/ *Yes*<br>☐ Tidak/ *No*
d. Pinjam daripada ahli keluarga atau kawan yang bukan isirumah<br>*Borrowed from non-household members or friends*	☐ Ya/ *Yes*<br>☐ Tidak/ *No*

e. Pinjam daripada bank/ ceti/ ah long dan lain-lain, selain kawan atau keluarga<br>*Borrowed from bank/ money lender and others, besides friends or family members*	☐ Ya/ *Yes*<br>☐ Tidak/ *No*
f. Lain-lain, sila nyatakan.....<br>*Others, please specify......*	....

D. Kos Tidak Langsung (Kehilangan Produktiviti)/ *Indirect cost (Productivity loss)*

Bahagian I diisi oleh pesakit yang **masih bekerja**/ *to be filled by those employed*

Soalan berikutnya adalah berkaitan dengan kos produktiviti yang hilang (tidak hadir bekerja) bagi tempoh **1 bulan yang lepas** disebabkan oleh masalah fizikal
*The following questions refer to your productivity loss (absent from work) for the **past 1 month** due to physical problem*

1. Berapa hari anda bekerja dalam seminggu?
   How many days a week do you work?
   a) Semasa/current …………….. hari/ *days.*
   b) Sebelum diagnosis barah/ prediagnosis …………….. hari/ *days.*

2. Adakah anda tidak hadir bekerja dalam tempoh 1 bulan yang lepas kerana sakit?
   Have you missed work in the last 1 month as a result of being sick?

   ☐ Tidak/ *No*
   ☐ Ya/ *Yes,*

   Saya tidak hadir bekerja selama ……. hari / *I have missed work for ……. days*

3. Pernahkah anda tidak hadir bekerja dalam tempoh sebelum 1 bulan yang lepas kerana sakit yang disebabkan oleh diagnosis barah anda?
   Did you miss work earlier than the period of 1 month due to illness related to your cancer diagnosis?

   ☐ Tidak/ *No*
   ☐ Ya/ *Yes,*

   Saya tidak hadir bekerja selama ……. hari / *I have missed work for ……. days*

4. Dalam tempoh 1 bulan yang lepas adakah terdapat hari bekerja anda yang telah diganggu disebabkan oleh masalah fizikal/ masah psikologi?
   During the last 4 weeks of your working days, have there been days in which you were bothered by physical or psychological problems?

   ☐ Tidak/ *No*
   ☐ Ya/ *Yes,*

   Jika tanda "ya", sila jawab soalan 5/ *if you tick "yes", please answer question 5*

5. Berapa hari bekerja anda yang telah diganggu oleh masalah fizikal atau masalah psikologi?
How many days at work, were you bothered by physical or psychological problems?

………………….. hari/ *days*

Bahagian II diisi oleh pesakit yang **tidak bekerja**/ ***Part B*** *is to be filled by unemployed*

Soalan berikutnya adalah berkaitan dengan kos produktiviti yang hilang (tidak dapat melakukan rutin harian) bagi tempoh **1 bulan yang lepas** disebabkan oleh masalah fizikal. Contohnya, anda menghadapi kesukaran untuk menjaga anak, menjalankan tugas di rumah, berkebun dan lain-lain
The following questions refer to your productivity loss (unable to do routine works) for the ***past 1 month*** *due to physical problem. For example, you have trouble caring for your children, unable to run errands, work in the garden or others.*

1. Dalam tempoh 1 bulan yang lepas, adakah terdapat hari yang anda terpaksa melakukan kurang kerja dari biasa disebabkan oleh masalah fizikal atau masalah psikologi?
For the past 1 months, were there days in which you were forced to do less unpaid work because of the physical or psychological problem?

☐ Tidak/ *No*
☐ Ya/ *Yes,*

2. Berapa hari keadaan ini berlaku? (dalam 1 bulan yang lepas)
How many days did this happen? (in the last 1 month)

………………….. hari/ *days*

3. Berapa jam dalam sehari anda kehilangan produktiviti?
How many hours in a day you are less productive?

………………….. jam/ *hours*

E. **Perbelanjaan isirumah/ *Household expenditure***<br><br>Soalan berikutnya adalah berkaitan dengan jumlah wang isirumah ini belanja dalam tempoh **1 bulan yang lepas**<br>*The following questions refer to the amount your household spends in the* ***last 1 month***	
Dalam tempoh sebulan lepas, berapa isirumah ini belanja untuk:<br>*In the last one month, how much did your household spend on:*	
7. Makanan, seperti beras, daging, buah-buahan, sayur-sayuran dan minyak masak. Ini termasuk harga sebarang makanan yang dimasak dan dimakan oleh isirumah, kecuali bayaran makan di restoran, arak dan tembakau<br>*Food, such as rice, meat, fruits, vegetables and cooking oils. This includes the cost of any food that was cooked and consumed by the household, excluding restaurant meals, alcohol and tobacco*	RM…………………………….
8. Sewa/ bayaran pinjaman rumah, bil elektrik, air dan telefon, gas dan arang/ kayu api<br>*Rental/ housing loan, electricity, water and telephone bill, gas and charcoal/ firewood*	RM………………………………
9. Yuran pendidikan (sekolah/ kolej/ tuisyen), buku, alat tulis dan lain-lain perbelanjaan berkaitan<br>*Education fees (school/ college/ tuition), books, stationeries, and other related expenses*	RM………………………………
10. Perbelanjaan untuk kesihatan, termasuk bayaran rawatan perubatan, ubat dan suplemen (tidak termasuk tuntutan insurans kesihatan)<br>*Healthcare expenses, including medical treatment fee, medicines and supplements (excluding health insurance reimbursement)*	RM……………………………….
11. Bayaran untuk insurans bagi jagaan kesihatan<br>*Payment for insurance for healthcare*	RM………………………………
12. Lain-lain perbelanjaan (bayaran ansuran dan minyak kereta/ motosikal, tol, tambang pengangkutan, pinjaman peribadi, bayaran makan di restoran, arak, tembakau, lampin dan susu bayi, dan lain-lain)<br>*Other expenses (instalment and fuel for car/ motorcycle, toll, transportation fee, personal loan, payment for meals at restaurants, alcohol, tobacco, diapers and milk for baby, and others*	RM…………………………….
13. Dalam tempoh sebulan yang lepas, secara keseluruhannya, berapakah yang telah dibelanjakan oleh isirumah anda?<br>*In the last one month, in total, how much did your household spend?*	RM………………………………
