# Supplementary material for: Cancer impact on lower-income patients in Malaysian public healthcare: An exploration of out-of-pocket expenses, productivity loss, and financial coping strategies
Source: PLoS One. 2024 Oct 9;19(10):e0311815. doi: 10.1371/journal.pone.0311815 (PMC11463769; doi:10.1371/journal.pone.0311815)
Supplement: S2 Appendix — All costs are reported in MYR; *Mann-Whitney U test; **Kruskal-Wallis test. (PDF) [file pone.0311815.s002.pdf]

S2 Appendix. Distribution of total OOP cancer costs by sociodemographic profiles.

Variables	Category	Median (IQR)	Mean (SD)	*p*-value
Age (years)	40-49	5,515.20 (6,698.50)	6,935.50 (6,515.67)	0.473**
	50-59	4,112.00 (4,915.75)	6,061.35 (7,144.89)	
	60-69	4,915.70 (6,034.00)	7,621.56 (10,285.75)	
	≥70	4,301.40 (8,489.70)	9,059.37 (14,418.82)	
Gender	Male	4,653.20 (5,721.94)	7,434.07 (10,390.49)	0.831*
	Female	4,657.60 (6,215.00)	7,150.33 (9,397.04)	
Ethnicity	Malay	4,920.00 (5,635.76)	6,249.88 (5,582.66)	0.845**
	Chinese	4,262.00 (8,234.20)	9,690.61 (15,631.33)	
	Indian	3,989.00 (5,268.70)	5,705.07 (5,000.03)	
	Indigenous Sabah	5,220.00 (6,627.20)	6,805.14 (5,310.25)	
	Indigenous Sarawak	4,240.00 (5,160.00)	5,791.74 (4,604.71)	
	Others	7,802.74	7,802.74 (4,755.63)	
Region	Peninsular Malaysia	4,784.60 (5,635.75)	7,750.33 (11,159.99)	0.650*
	Sabah and Sarawak	4,380.00 (6,461.75)	6,133.27 (5,271.05)	
Marital Status	Married	4,657.60 (6,137.50)	7,384.34 (10,122.09)	0.536**
	Divorced/ separated/ widow	4,911.40 (4,967.80)	6,140.32 (5,515.95)	
	Single	3,489.76 (8,184.49)	7,747.48 (11,426.15)	
Education Level	Primary level	4,526.60 (5,256.75)	7,365.49 (11,392.89)	0.785**
	Secondary level	4,915.70 (6,281.63)	7,160.52 (9,149.77)	
	Tertiary level	4,544.50 (5,502.71)	7,768.97 (8,856.89)	
	No formal education	2,950.00 (7,140.14)	7,030.49 (8,903.92)	
Employment	Employed	5,440.00 (7986.10)	9,365.19 (14,828.20)	0.162**
	Unemployed	4,765.00 (6113.55)	7,283.24 (9,364.38)	
	Retiree	3,600.00 (4005.30)	5,114.05 (5,386.06)	
Monthly household income (MYR)	<2000	4,000.00 (5142.80)	5,594.97 (6,248.71)	0.060**
	2000-4500	4,961.00 (6943.85)	7,873.01 (10,411.18)	
	>4500	4,744.00 (6622.30)	8,721.84 (12,430.02)	
Diagnosis	Breast	4,168.00 (5,938.20)	6,685.20 (8,217.19)	0.285**
	Respiratory	4,178.00 (5,563.01)	6,592.86 (9,207.96)	
	Gastrointestinal	5,011.60 (6,490.75)	8,637.97 (11,867.73)	
	Urogenital	3,068.00 (4,815.00)	9,378.06 (19,803.78)	
	Female reproductive	4,680.00 (6,938.40)	6,586.21 (6,101.75)	
	Others	4,920.00 (5,373.68)	6,469.83 (5,695.35)	
Cancer stage	Stage 1	3,069.80 (7,380.00)	5,389.90 (4,280.38)	0.099**
	Stage 2	3,480.00 (5,452.80)	7,041.85 (12,810.00)	
	Stage 3	4,920.00 (5,584.20)	6,927.40 (8,284.25)	
	Stage 4	4,720.00 (6,298.10)	7,719.27 (9,843.46)	

All costs are reported in MYR
*Mann-Whitney U test; **Kruskal-Wallis test
