# Supplementary material for: Cancer impact on lower-income patients in Malaysian public healthcare: An exploration of out-of-pocket expenses, productivity loss, and financial coping strategies
Source: PLoS One. 2024 Oct 9;19(10):e0311815. doi: 10.1371/journal.pone.0311815 (PMC11463769; doi:10.1371/journal.pone.0311815)
Supplement: S3 Appendix — ΨCovariates = the continuous variables; *Variables with p<0.25 were fitted into the final model. (PDF) [file pone.0311815.s003.pdf]

Variables	Category	Exp(β)-coefficient	95% CI	*p*-value
Age	40-49	Ref	Ref	Ref
	50-59	0.87	0.66 – 1.15	0.339
	60-69	1.10	0.84 – 1.44	0.489
	≥70	1.31	0.95 – 1.80	0.101*
Gender	Male	Ref	Ref	Ref
	Female	0.96	0.80 – 1.16	0.678
Ethnicity	Malay	Ref	Ref	Ref
	Chinese	1.55	1.26 – 1.91	<0.001*
	Indian	0.91	0.67 – 1.25	0.566
	Indigenous Sabah	1.09	0.81 – 1.47	0.578
	Indigenous Sarawak	0.93	0.67 – 1.27	0.639
	Others	1.25	0.35 – 4.47	0.733
Marital Status	Married	Ref	Ref	Ref
	Divorced/ separated/ widow	0.83	0.64 – 1.09	0.174*
	Single	1.05	0.76 – 1.46	0.775
Education Level	Primary level	Ref	Ref	Ref
	Secondary level	0.97	0.79 – 1.19	0.786
	Tertiary level	1.06	0.73 – 1.52	0.774
	No formal education	0.96	0.66 – 1.34	0.807
Employment	Employed	Ref	Ref	Ref
	Unemployed	0.78	0.58 – 1.04	0.088*
	Retiree	0.55	0.38 – 0.80	0.002*
Household size	1-4	Ref	Ref	Ref
	5-8	1.04	0.87 – 1.26	0.664
	≥9	0.87	0.52 – 1.46	0.594
Monthly household income	MYR <2000	Ref	Ref	Ref
	MYR 2000-5000	1.41	1.15 – 1.72	<0.001*
	MYR >5000	1.56	1.23 – 1.97	<0.001*
Region	Peninsular Malaysia	Ref	Ref	Ref
	Sabah & Sarawak	0.79	0.65 – 0.96	0.016*
Diagnosis (cancer site)	Breast	Ref	Ref	Ref
	Respiratory	0.99	0.73 – 1.34	0.929
	Gastrointestinal	1.29	1.02 – 1.64	0.033*
	Urogenital	1.40	0.93 – 2.11	0.106*
	Female reproductive	0.99	0.72 – 1.34	0.924
	Others	0.97	0.74 – 1.26	0.810
Cancer stage	Stage 1	Ref	Ref	Ref
	Stage 2	1.31	0.81 – 2.12	0.278
	Stage 3	1.29	0.81 – 2.03	0.283
	Stage 4	1.43	0.91– 2.25	0.118

Time since diagnosis	<1 year	Ref	Ref	Ref
	≥1 year	0.81	0.68 – 0.96	0.017*
Present treatment	Chemotherapy	Ref	Ref	Ref
	Radiotherapy	1.02	0.78 – 1.32	0.904
	Surgery	1.59	0.88 – 2.85	0.123*
	Chemotherapy + Radiotherapy	1.16	0.78 – 1.72	0.457
	Others	1.02	0.78 – 1.32	0.737
Number of inpatient visits/ year$^{\Psi}$	-	1.02	0.98 – 1.07	0.396
Number of admission days/ year$^{\Psi}$	-	1.00	1.00 – 1.01	0.264
Number of outpatient visits/ year$^{\Psi}$	-	1.00	1.00 – 1.01	0.028*
Distance to cancer centre$^{\Psi}$	-	1.00	0.99 – 1.00	0.628
